# Proteins from *Tuber magnatum *Pico fruiting bodies naturally grown in different areas of Italy

**DOI:** 10.1186/1477-5956-11-7

**Published:** 2013-02-01

**Authors:** Federico Vita, Valentina Lucarotti, Emanuele Alpi, Raffaella Balestrini, Antonietta Mello, Angela Bachi, Massimo Alessio, Amedeo Alpi

**Affiliations:** 1 Department of Crop Plant Biology, University of Pisa, via Mariscoglio 34, 56124 Pisa, Italy; 2 Biomolecular Mass Spectrometry Unit, San Raffaele Scientific Institute, Milan, Italy; 3 Proteome Biochemistry Unit, San Raffaele Scientific Institute, Milan, Italy; 4 Plant Protection Institute, Turin UOS, CNR, Viale Mattioli, 25, 10125, Turin, Italy

**Keywords:** Tuber magnatum, Proteins, Fruiting bodies, 2D-electrophoresis, Mass spectrometry, qPCR

## Abstract

**Background:**

A number of *Tuber* species are ecologically important. The fruiting bodies of some of these also have value as a cooking ingredient due to the fact that they possess exceptional flavor and aromatic properties. In particular, *T. magnatum* fruiting bodies (commonly known as truffles), are greatly appreciated by consumers. These grow naturally in some parts of Italy. However, the quality of these fruiting bodies varies significantly depending on the area of origin due to differences in environmental growth conditions. It is therefore useful to be able to characterize them. A suitable method to reach this goal is to identify proteins which occur in the fruiting bodies that are specific to each area of origin. In this work protein profiles are described for samples coming from different areas and collected in two successive years. To our knowledge this is the first time that proteins of *T. magnatum* have been thoroughly examined.

**Results:**

Using two dimensional electrophoresis, reproducible quantitative differences in the protein patterns (total 600 spots) of samples from different parts of Italy (accession areas) were revealed by bioinformatic analysis. 60 spots were chosen for further analysis, out of which 17 could probably be used to distinguish a sample grown in one area from a sample grown in another area. Mass spectrometry (MS) protein analysis of these seventeen spots allowed the identification of 17 proteins of *T. magnatum.*

**Conclusions:**

The results indicate that proteomic analysis is a suitable method for characterizing those differences occurring in samples and induced by the different environmental conditions present in the various Italian areas where *T. magnatum* can grow. The positive protein identification by MS analysis has proved that this method can be applied with success even in a species whose genome, at the moment, has not been sequenced.

## Background

The ectomycorrhizal fungus *Tuber magnatum* Pico is an hypogeous ascomycete that forms specialized symbioses with fine roots of higher plants. The interest on this specific *Tuber* is undoubtedly related to its ecological role in boreal and temperate forests, though the use of its fruiting bodies (commonly known as truffles) as a cooking ingredient is certainly equally relevant. Truffles are greatly appreciated for their intense flavor and aroma. [1]. Several attempts have been made to artificially grow this fungus, which to date have been unsuccessful. As a result, the fruiting bodies must be obtained from their natural place of growth, in the woods. Knowing where to look for truffles requires years of training and experience, and often requires the help of specially trained animals, for example dogs. In addition, the fruiting bodies grow only in a restricted number of locations and environments. These difficulties, together with the high demand for truffles on the market, drive up their price significantly.

The quality of the fruiting bodies can be affected by their place of origin. A reliable method to differentiate between truffles from different locations – and thus reassure to consumers as to the quality of the product they will pay a high price for – has long been sought.

*T. magnatum* is present in some parts of Italy (Tuscany, Piedmont, Marche, Umbria, etc.), Istria and in several Balkan regions. The quality of its fruiting bodies is variable and depends on the environment and the growth conditions. Due to limited availability and high market value, *T. magnatum* is the subject to fraudulent claims [2]. Gene expression, and consequently the protein profile, is strongly influenced by many factors. In addition to the conditions in the location where the fungus grows, these include the developmental stage of the fungus itself, and small genetic variations. This last factor has been recently shown to correlate with variability in volatile compound production in *T. aestivum*[3].

The life cycle of a truffle can be divided into stages [4] and many attempts have been made to characterize and to distinguish them in species that are very similar from a morphological point of view (e.g., white truffles group). To reach this goal conventional classification methods have been used, as well as more sophisticated analytical tools. Polymerase chain reaction (PCR) has been used to try to identify molecular markers suitable for distinguishing truffle species during their life cycle [5]. The use of Simple Sequence Repeat (SSR) also showed the possibility to use a molecular marker to trace the *T. magnatum* life cycle [6]. The results are encouraging, but different environmental origins of the fruiting bodies cannot be determined using these methods.

A proteomic approach to resolving this problem therefore appeared suitable. Initial attempts were made by French groups [7] using fruiting body protein analysis as a taxonomic criterion in superior mushrooms. This method made it possible to differentiate several *Tuber* species. Later these investigations were extended to other French and Italian samples, again with the aim of distinguishing between species [8]. These results are also encouraging although most efforts were then concentrated on isoenzyme analysis [9] and the problem of differentiating areas of origin within the same species remains unresolved. High-resolution two-dimensional gel electrophoresis (2-DE) has been used in several studies to generate fungal protein maps [10]. Protein variability may originate from alternative splicing, post-translational modifications, and amino and carboxy-terminal modifications. Also, the formation of disulfide bonds, glycosylation, the addition of lipid groups, and partial proteolysis can vary. [11]. The interaction between a particular environment and the genome can generate different proteins, different protein structures (qualitative differences) or proteins in varying amounts (quantitative differences). Recently the haploid genome of *Tuber melanosporum* has been sequenced [4], marking a step forward in understanding of the biology and evolution of ectomycorrhizal symbiosis. The identification of proteins of this species, as well as in others in the same genus, has been significantly facilitated.

Based on these considerations, we have used two dimensional electrophoresis; image and statistical analysis; mass spectrometry; database search and protein blast, to compare protein profiles of fruiting bodies of *T. magnatum* Pico, naturally grown in different Italian areas, with the aim of characterizing them. In this first contribution some proteins that could enable to distinguish origin of fruiting bodies are reported, while a thorough characterization of them would follow in the near future.

## Results and discussion

**Figure 1:**
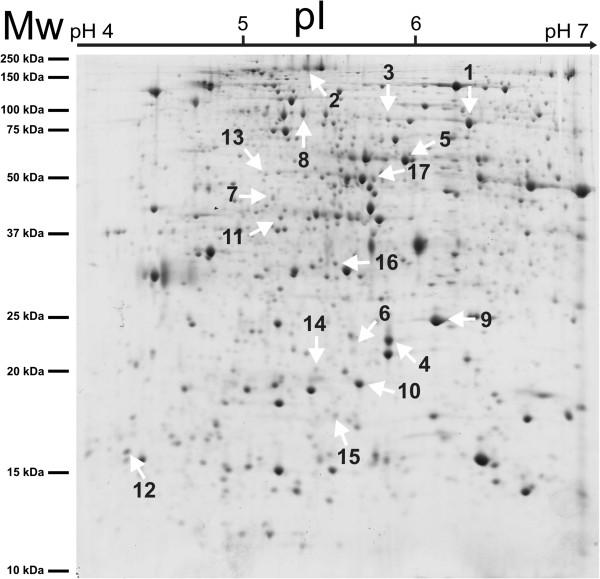
**Representative 2D gel from the San Miniato sample (1 mg of protein extract) stained with colloidal Coomassie. **Spots selected by bioinformatic analysis to discriminate between different area of origin as shown here.

**Figure 2:**
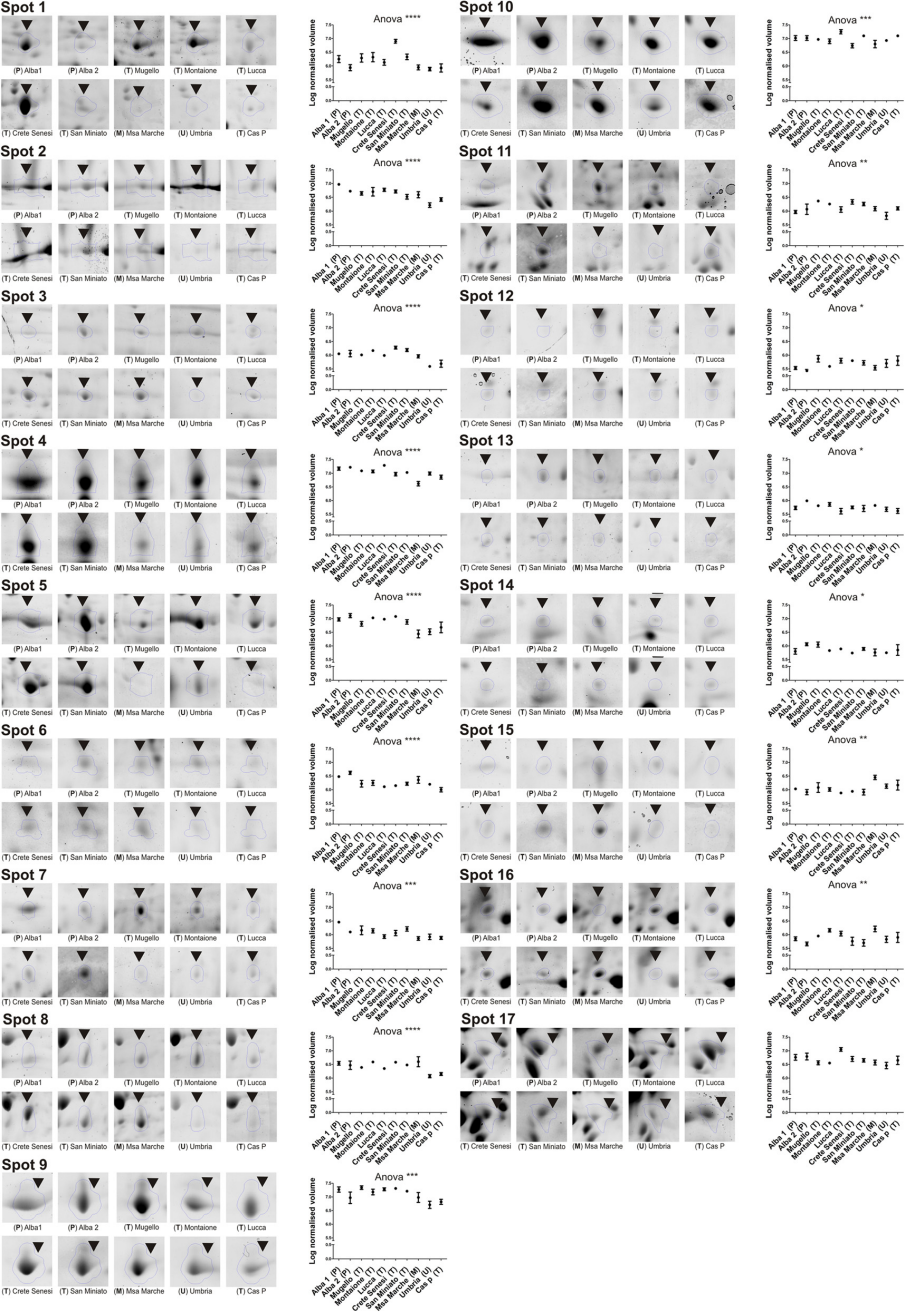
**Intensity levels of the spots selected for MS analysis. **Enlarged areas from 2D-E representative gels are shown and the relative quantitation (n= 6) are reported as values expressed in log_10 _normalized volume (spot optical density) with ± SE. Statistical significance evaluated by ANOVA is reported as p-values. *= p-value < 0,05, ** = p-value < 0,01, *** = p-value < 0,001, **** = p-value < 0,0001. T= Tuscany, P= Piedmont, U= Umbria, M= Marche.

Samples collected in two years (as described in Methods -section "fruiting bodies") from different parts of Italy were used for proteomic analysis. The degree of maturation of fruiting bodies was measured according to [12] and reported as stage 5, with the presence of 80-100% mature spores that were yellow-reddish brown in color with reticulate ornamentation [13]. At least six independent replicate gels (three for each year analyzed) for all accessions of *Tuber* were performed. Gels were analyzed for the number of spots, and the intensity and size of the spots. Over 600 reproducible protein spots were detected in each gel. The comparison of protein patterns in different accessions of *Tuber* revealed the presence of quantitative differences (number of spots) that remained constant in different collecting years (Figure 1, Figure 2).

**Figure 3:**
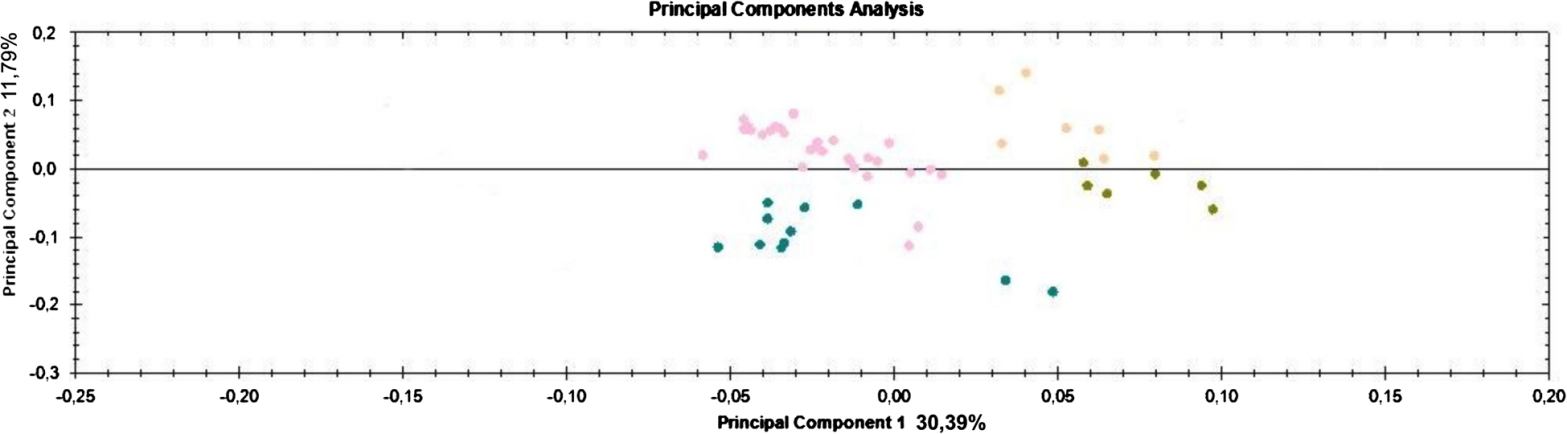
**Principal component Analysis obtained on the base of the ability of the 17 spots to discriminate the area of origin. **Pink circles represent Tuscany’s samples; Green olive circles represent Umbria samples; Yellow circles represent Marche samples; Blue circles represent Piedmont samples.

**Figure 4:**
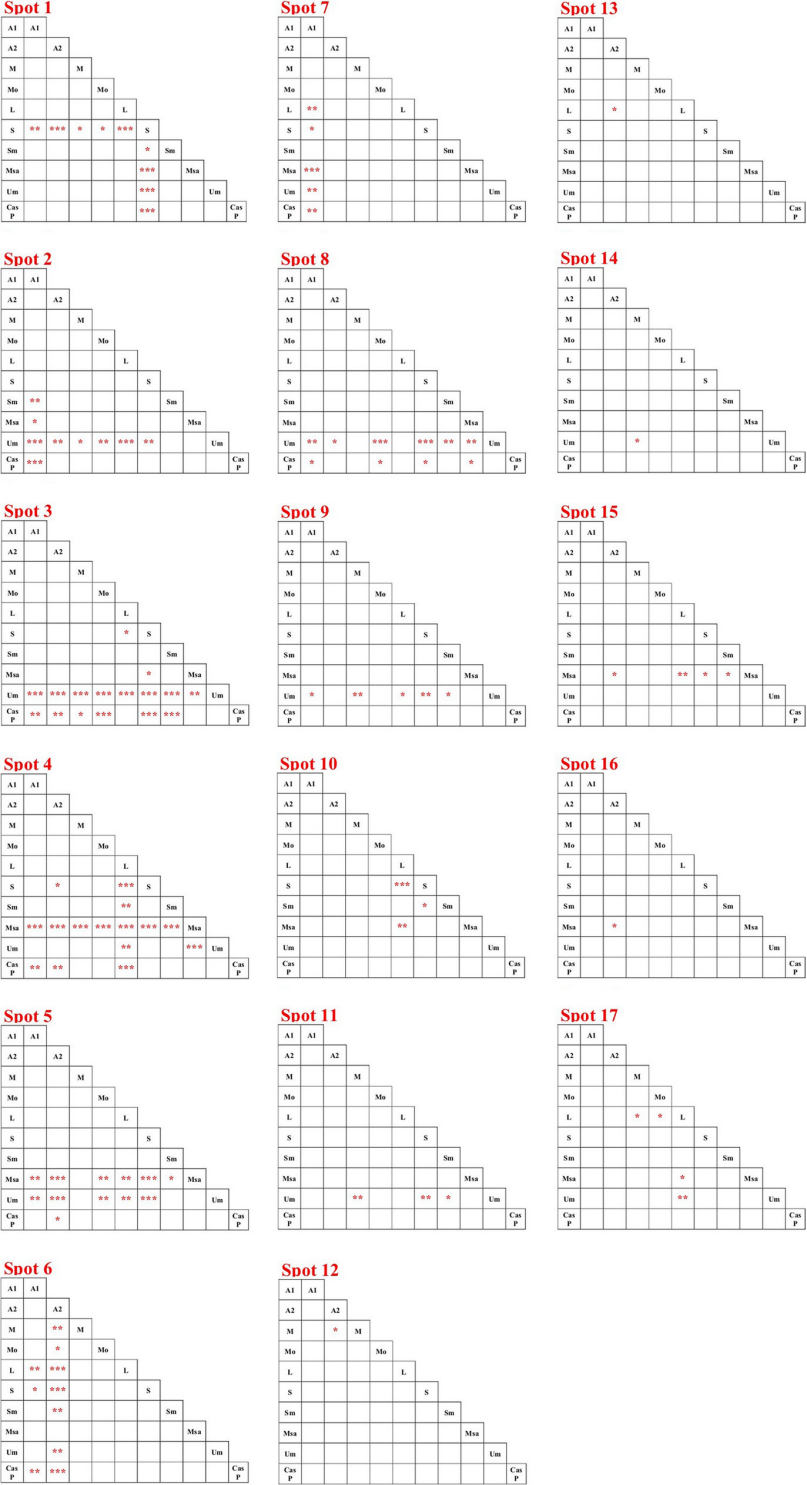
Tukey post test results, performed on the base of the ANOVA results, for the comparison of samples’s pairs. *= p value < 0,05, ** = p-value < 0,01, *** = p-value < 0,001, **** = p-value < 0,0001; A1 = Alba, A2 = Alba 2, M = Mugello, Mo = Montaione, L = Lucca, S = Crete Senesi, Sm = San Miniato, Msa = Marche, Um = Umbria, Cas P = Casentino.

After bioinformatic analysis, 60 spots showed statistically significant values of ANOVA and fold change which potentially could provide a basis to distinguish between samples. Among these, seventeen were most suitable to describe the area of origin, as assessed by principal component analysis (PCA) and were therefore selected for MS analysis (Figure 1, Figure 2, Figure 3). The PCA results (Figure 3), showed that it is possible to distinguish between samples from different regions (Tuscany, Piedmont, Marche, Umbria) by examining on the expression level of these 17 spots. As it is shown in Figure 2 the difference in expression level of specific spots contribute to the generation of cluster. For instance spots 2 and 8 strongly contribute to form a cluster that include the Umbria samples, being the average normalized intensity values lower than the other accession areas (Figure 2), while the spots 6 and 7 were able to distinguish samples coming from Piedmont (Alba 1 and 2). On the other hand, the sample collected in the Marche region on the other hand, may be identified by spot 12, while the accessions from Tuscany (the largest group), showed consistently differences for spots 3 and 5 (Figure 2). Furthermore, a post-analysis test for the ANOVA results comparing pairs of samples (Figure 4) showed that some samples are differentiable within the area of origin (namely Tuscany). For example, the sample Crete Senesi, presents the differences of expression in spots 1 and 3, while spots 4 and 10 distinguish the sample Lucca from the others.

Mass spectrometry analysis allowed to get a protein sequence for 15 out of 17 spots. Two spots (10 and 12) failed to give protein identifications while spots 5, 13, 16 and 17 gave rise to the identification of two different proteins in each spot. 17 different proteins were identified (16 through ESI-TOF analysis and 1 by MALDI analysis) even though in spots 2 and 8, and 6 and 9 different isoform of the same proteins were identified (Table 1, Table 2). All of the proteins, with the exception of Q1ACW3 (NADP-dependent mannitol dehydrogenase) had been previously found in *T. melanosporum* (Table 1). To ensure a putative functional identification, protein blast (UniprotKB) was performed when no other information was available.

**Table 1:** Proteins identified by ESI-Quad TOF and MALDI analysis

**ESI**														
**Spot No. (a)**	**Acc. No. (b)**	**Organism**	**Protein Description**	**Score (c)**	**Seq. Cov. % (d)**	**Mw kDa Obs/Theo (e)**		**pI Obs/Theo (f)**		**Identified peptides (MS/MS) (g)**	***Tuber *Gene annotation (h)**	**KOG Definition (i)**	**PFAM Definition (l)**	**Protein Blast relation (m)**
**1**	D5G797	*T. melanosporum *(Perigord truffle)	Whole genome shotgun sequence assembly, scaffold_131, strain Mel28	104	4	78	46,2	6,2	5,82	2	**GSTUM_00002523001**	Aminoacylase ACY1	Peptidase M20	**A**
**2**	D5GJY5	*T. melanosporum *(Perigord truffle)	Whole genome shotgun sequence assembly, scaffold_55, strain Mel28	378	11	162	86,3	5,4	5,16	7	**GSTUM_00009270001**	Ultrahigh sulfur keratin-associated protein	Glyoxal oxidase	**B**
**3**	D5GGN7	*T. melanosporum *(Perigord truffle)	Dihydrolipoyl dehydrogenase	137	6	81	54	5,7	6,72	3	**GSTUM_00007439001**	Dihydrolipoamide dehydrogenase	Pyridine nucleotide-disulphide oxidoreductase	
**4**	D5G8F0	*T. melanosporum *(Perigord truffle)	Whole genome shotgun sequence assembly, scaffold_15, strain Mel28	164	28	22,5	20	5,7	5,41	4	**GSTUM_00004791001**	Peptide methionine sulfoxide reductase	Peptide methionine sulfoxide reductase	**C**
**5**	D5GJ78	*T. melanosporum *(Perigord truffle)	S-adenosyl methionine synthase	871	47	57	41,8	5,8	5,7	15	**GSTUM_00008874001**	S-adenosylmeth. synthetase	S-adenosylmeth. synthetase	
	D5G9M7	*T. melanosporum *(Perigord truffle)	Whole genome shotgun sequence assembly, scaffold_169, strain Mel28	114	12	57	57,8	5,8	9,93	5	**GSTUM_00003332001**	RNA-binding protein	RNA recognition motif	**D**
**6**	D5GNA2	*T. melanosporum *(Perigord truffle)	Whole genome shotgun sequence assembly, scaffold_8, strain Mel28	125	22	22	22	5,5	6,21	4	**GSTUM_00011192001**	1,4-benzoquinone reductase	Flavodoxin	**E**
**7**	D5GAF9	*T. melanosporum *(Perigord truffle)	Whole genome shotgun sequence assembly, scaffold_18, strain Mel28	146	8	45	33	5,1	6,02	4	**GSTUM_00005271001**	Stationary phase-induced protein	SOR/SNZ family	**F**
**8**	D5GJY5	*T. melanosporum *(Perigord truffle)	Whole genome shotgun sequence assembly, scaffold_55, strain Mel28	170	9	91	86,3	5,4	5,16	6	**GSTUM_00009270001**	Ultrahigh sulfur keratin-associated protein	Glyoxal oxidase	
**9**	D5GNA2	*T. melanosporum *(Perigord truffle)	Whole genome shotgun sequence assembly, scaffold_8, strain Mel28	1206	35	25	22	6,1	6,21	6	**GSTUM_00011192001**	1,4-benzoquinone reductase	Flavodoxin	**G**
**11**	D5G6L6	*T. melanosporum *(Perigord truffle)	Whole genome shotgun sequence assembly, scaffold_121, strain Mel28	118	21	41	31,3	5,2	6,01	5	**GSTUM_00002097001**	Predicted dehydrogenase	Short chain dehydrogenase	
**13**	D5GC43	*T. melanosporum *(Perigord truffle)	Whole genome shotgun sequence assembly, scaffold_201, strain Mel28	149	18	38	33,5	5.1	5,06	5	**GSTUM_00000555001**	Lysophosphatidic acid acyltransferase	BAR domain protein	**H**
	D5GAC6	*T. melanosporum *(Perigord truffle)	Probable Xaa-Pro aminopeptidase	131	5	38	69,2	5.1	5,28	3	**GSTUM_00005237001**	Xaa-Pro aminopeptidase	Peptidase_M24	
**14**	D5GCN2	*T. melanosporum *(Perigord truffle)	Whole genome shotgun sequence assembly, scaffold_218, strain Mel28	83	22	19	20,7	5.4	8,64	3	**GSTUM_00000743001**	Alkyl hydroperoxide reductase/peroxiredoxin	Redoxin	**I**
**15**	D5G620	*T. melanosporum *(Perigord truffle)	Malate dehydrogenase	115	8	18	36,7	5.5	8,79	3	**GSTUM_00001731001**	NAD-dependent malate dehydrogenase	Lactate/malate dehydrogenase	
**16**	D5GE86	*T. melanosporum *(Perigord truffle)	Whole genome shotgun sequence assembly scaffold_26, strain Mel28	105	14	28,2	26,8	5.5	7,1	4	**GSTUM_00006427001**	Predicted hydrolases of HD superfamily	No Hit Found	**L**
**17**	D5G5R4	*T. melanosporum *(Perigord truffle)	Whole genome shotgun sequence assembly, scaffold_112, strain Mel28	376	11	45,6	46,9	5.7	5,97	4	**GSTUM_00001447001**	Fructose 1,6-bisphosphate aldolase	Fructose-bisphosphate aldolase	**M**
	Q1ACW3	*Tuber borchii*	NADP-dependent mannitol dehydrogenase	314	26	45,6	38	5.7	5,69	7	**DQ223686.1**	NAD-dependent malate dehydrogenase	NAD-dependent malate dehydrogenase	
**MALDI**														
**Spot No. (a)**	**Acc. No. (b)**	**Organism**	**Protein Description**	**Score (c)**	**Seq. Cov. % (d)**	**Mw kDa Obs/Theo (e)**		**pI Obs/Theo (f)**		**Identified peptides (MS) (g)**	***Tuber *Gene annotation (h)**	**KOG Definition (i)**	**PFAM Definition (l)**	**Protein Blast relation (m)**
**16**	D5GJE0	*T. melanosporum *(Perigord truffle)	Whole genome shotgun sequence assembly, scaffold_50, strain Mel28	80	40%	28.2	25.6	5.5	5.77	11	**GSTUM_00008955001**	Alkyl hydroperoxide reductase	AhpC-TSA, AhpC/TSA family	

Table 1 List of proteins identified by ESI-Quad TOF and MALDI analysis. (a) as indicated in Figure 1; (b) UniProtKB accession number; (c) Mascot score; (d) percent sequence coverage; (e) gel-observed vs. theoretical molecular weights * Observed and theoretical weight may differ on the base of the real molecular weight of the protein in *T. magnatum*; (f) observed vs. theoretical isoelectric points; (g) number of matched peptides; (h) gene reference code; (i) and (l), gene model annotations (from http://mycor.nancy.inra.fr/IMGC/TuberGenome/Search.html); (m) numerical correlation to proteins obtained by blast analysis (Table 2). Spot 10 and Spot 12, no protein identification.

**Table 2:** Additional information on the proteins obtained by blast analysis

**Spot No. (a)**	**Protein Blast relation (b)**	**Acc. No. (c)**	**Protein Name**	**Organism**	**Protein Length (d)**	**E-Value (e)**
1	**A**	G2YT91	**Similar to peptidase (Secreted protein)**	*Botryotinia fuckeliana*	383	1.0×10^-120^
2, 8	**B**	B2WA62	**Glyoxal oxidase**	*Pyrenophora tritici-repentis*	825	0.0
4	**C**	B2WK75	**Peptide methionine sulfoxide reductase msrB/msrA**	*Pyrenophora tritici-repentis*	184	3.0×10^-96^
5	**D**	Q4WLU4	**RNP domain protein**	*Neosartorya fumigata*	480	1.0×10^-120^
6, 9	**E**	C4JW16	**NAD(P)H:quinone oxidoreductase, type IV**	*Uncinocarpus reesii*	203	1.0×10^-100^
7	**F**	E5ABQ9	**Similar to pyridoxine biosynthesis protein**	*Leptosphaeria maculans*	307	1.0×10^-176^
11	**G**	C5FLT9	**3-oxoacyl-[acyl-carrier-protein] reductase**	*Arthroderma otae*	289	2.0×10^-92^
13	**H**	A4D9A0	**BAR protein**	*Neosartorya fumigata*	305	1.0×10^-137^
14	**I**	B8MCW9	**AhpC/TSA family protein**	*Talaromyces stipitatus*	181	3.0×10^-50^
16	**L**	C1G7T9	**HD domain-containing protein**	*Paracoccidioides brasiliensis*	224	4.0×10^-62^
17	**M**	G0RU75	**Fructose bisphosphate aldolase**	*Hypocrea jecorina*	360	0.0

Table 2 Protein Blast results of the identified proteins mentioned in Table 2. (a) as indicated in Figure 1; (b) alphabetical correlation to proteins previously reported (Table 2); (c) UniProtKB, accession number; (d) as reported from blast output; (e) Expect value, the lower the E-value the higher is the “significance” of the match.

Some of the proteins identified can be grouped according to the metabolic pathways that they belong to. Spots 4 and 5 belong to the methionine metabolism. Spot 5 (Table 1, Table 2) is a S-adenosylmethionine synthetase; this enzyme, similarly to the peptide methionine sulfoxide reductase (spot 4, Table 1, Table 2), could function protecting cell against oxidative damage [14]. S-adenosylmethionine synthase appears to take part in cystein/methionine biosynthesis and interconversion, playing a key role in the production of hydrogen sulfide [4]. Hydrogen sulfide is a precursor of many volatile compounds, two of which, dimethyl trisulfide and dimethyl disulfide [15], are among the main volatile compounds responsible for the *T. magnatum* flavor. Other proteins can be grouped on the basis of their redox activity. Dihydrolipoyl dehydrogenase (spot 3), plays a role in cell redox homeostasis, while glyoxal oxidase (spot 2 and 8) catalyzes the oxidation of several aldehydes producing extracellular H_2_O_2_[16]. It is interesting to observe that glyoxal oxidase shares some traits with another fungal enzyme, galactose oxidase. The critical active site residues typical of radical copper oxidases are conserved between these two enzymes [17]. Galactose oxidase catalyzes the oxidation of primary alcohols to aldehydes and is reported to be a monomeric enzyme of 68.5 kDa [18], though previously was considered a dimer or higher polymer [19]. In our work glyoxal oxidase has been identified at two different molecular weights (91 and 162 kDa), although at the same pI. Also the intensity values are similar for both spots and correlate well with transcript data, as demonstrated by Alba 1 and Umbria samples that represent respectively the highest and lowest expression level.

**Figure 5:**
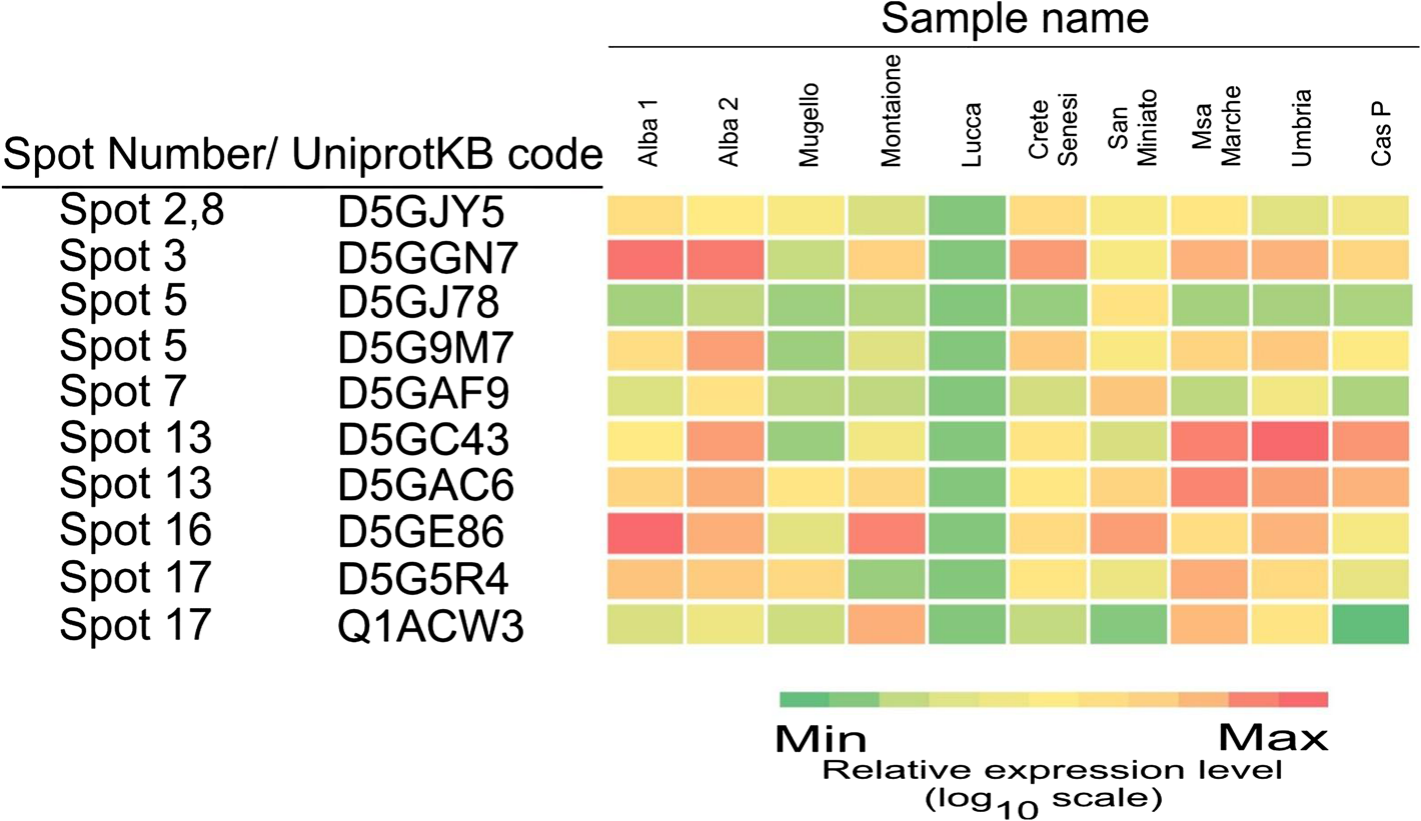
**Coordinate regulation of gene related proteins. **The Lucca sample was selected as internal control and it represents the less expressed sample. (n=4) Cas P = Casentino.

In order to obtain further data in addition to protein identification, we performed a gene expression analysis through qPCR on selected genes. Two technical replicates were performed for each qPCR analysis. When it was not possible to design specific primers (e.g. malate dehydrogenase) due to lack of information, no data were obtained. qPCR was also useful in the case of double protein identification, to try to determine which of the two is responsible for the expression changes observed between truffles of different origin. 10 out of 17 genes related to as many proteins were analyzed by this method. For the remaining 7 genes, it proved impossible to select an efficient set of primer due to the current lack of sequence information. The genes analyzed in the Lucca sample present low levels of expression when compared to all other samples, probably due to the quality of the RNA that was lower than in others. With the exception of this sample, the gene expression data generally correlated well with the related protein levels, as shown by spot 2 and 7 (Figure 2, Figure 5). In spot 2, both the protein levels of D5GJY5 (putative glyoxal oxidase, Table 1, Table 2) and the corresponding transcripts were highest in the Crete Senesi sample. The same was true for spot 7 (D5GAF9, putative pyridoxine biosynthesis protein, Table 1, Table 2), in which both protein and transcript levels were highest in the San Miniato sample. In four cases (spots 5, 13, 16 and 17) two proteins were identified for each spot. For these, both the relative transcripts were analyzed, except for spot 16. As shown in Figure 5, transcript levels for spot 5 (protein D5G9M7), fit the trend. Similarly in spot 13, the transcript level related to protein D5GAC6 is well correlated with the spot trend. This may suggest that those proteins are the ones that contribute the most to the difference of intensity detected in spot 5 and 13 .

## Conclusions

The aim of this research was to assess protein profiles of fruiting bodies of *T. magnatum* grown in two years from different areas of Italy. We harvested truffles, in successive years, from the same location (when possible, from the same plant). As shown in our results, we identified several proteins vary according to the place of origin, but that do not change from one year to the next. Although a complete characterization of the *T. magnatum* fruiting body proteome would require further work, to our knowledge, the proteins identified in this work represent the first contribution on this subject. This was achieved in part thanks to the recent sequencing of *T. melanosporum* which greatly improved the likelihood of correctly identifying proteins in an organism belonging to the same genus whose proteome has not yet been studied. It was the availability of this basic information which made possible the recognition of several proteins.

These proteins (reported in Table 1, Table 2) have biochemically distinct functions, only some of them belonging to the same pathway. Considering this, together with the very limited proteome coverage that our initial approach afforded, general biochemical or physiological comments could be somewhat preliminary. Also, we decided to MS-analyze only those spots that were statistically significant after image analysis and not all the entire proteome displayed on the gels, since they better characterized the different accession areas.

The fungus is an organism that is not autotrophic and hence relies on plant-originated carbohydrate breakdown in order to get energy for its metabolism. NADP-dependent mannitol dehydrogenase is linked to the carbohydrate metabolism [20]. This enzyme catalyzes the conversion of mannitol to mannose, but it can also be very active in the reversible conversion of fructose to mannitol [21]. It seems logical that fruiting bodies originated in different environments are modulated in the subtle though fundamental carbohydrate metabolism pathways.

Sugars are channeled to glycolysis and the TCA cycle to produce energy and metabolites. A TCA cycle enzyme (malate dehydrogenase) is one of the proteins affected by the environment giving support to the idea that the fungal respiratory metabolism is influenced. Alternatively this enzyme could be involved in the glyoxylate cycle and hence in the gluconeogenesis pathway [22] with its peculiar dual function.

Protein D5GJY5, identified as glyoxal oxidase with a good e-value after protein blast analysis, represents an interesting protein that is important for lignin degradation through production of extracellular H_2_0_2._[16,23]. On the other hand T. *magnatum* moves along differential nutritional strategies (saprotrophic, endophytic and symbiotic) depending on the environment and on the developmental phase of their life cycle [24].

For the remaining identified proteins it is more difficult to extrapolate specific physiological meanings, even though all of them have important biological functions.

Certainly with these results we have shown that several proteins of *T. magnatum*, a species whose genome at the moment is not still sequenced, could be identified and a specific biochemical role can be assigned to them; with more high-throughput proteomic analysis, planned for the future, a more definitive physiological frame could be available.

The results of this study show how it is possible to use a proteomic approach to verify the consistency of quantitative variation of proteins of interest. The results showed a high reproducibility of the patterns from samples collected in different years. We have individuated 17 proteins from *T. magnatum* Pico, which provide a basis for the future development of proteomic characterization of fruiting bodies of different origin.

## Methods

### Fruiting bodies

Fruiting bodies belonging to the various *T. magnatum* accessions were collected from natural ground in central and northern Italy (Tuscany, Piedmont, Marche and Umbria) in two successive years as reported in Table 3. At least three fruiting bodies were used for each biological replicate and their protein extracts were then mixed to increase the homogeneity of the sample. Fruiting bodies were thoroughly washed several times with distilled water and subsequently dipped in absolute ethyl alcohol to remove external contamination. Finally the thin external layer of the peridium was removed. Microbiological analysis, carried out to verify the presence of micro-organisms inside the gleba (inner tissue of the fruiting bodies), showed that after ethanol treatment the residual microbial contaminants were still present, but the number of CFU were reduced to such a low level that the detection of their proteins would not be possible (data not shown). Samples were frozen in liquid nitrogen and the tissue was ground in a mortar, then stored at -80°C before being used for protein analysis.

**Table 3:** Samples under analysis

**Accessions (Collection place)**	**Host Plant**	**Region**	**Collection period**
San Miniato	Wood	Tuscany	November 2008-2009
Crete Senesi	Wood	Tuscany	November 2008-2009
Mugello	*Tilia* sp.	Tuscany	November 2008-2009
Montaione	Wood	Tuscany	November 2008-2009
Lucca	(*Populus tremula*)	Tuscany	November 2008-2009
Umbria	Wood	Umbria	November 2010-2011
Alba 1	(*Populus tremula)*	Piedmont	November 2008-2009
Alba 2	(*Quercus ruber*)	Piedmont	November 2008-2009
Msa Marche	(*Populus tremula)*	Marche	November 2010-2011

Table 3 Samples collected in two years in different Italian areas. In four cases it was not possible to trace back the specific host plant. Msa Marche sample from Sant’Angelo in Vado (PU), Umbria samples from Corciano (PG). Fruiting bodies used for each year of analysis; 2008 (n=3), 2009 (n=4), 2010 (n=3), 2011 (n=3).

### Protein extraction

Fruiting bodies (100 mg) were ground in liquid nitrogen and homogenized with 1.6 mL of extraction buffer (Urea 8 M, Tris–HCl 40 mM CHAPS 4%, DTT 60 mM) according to [25] with some modifications. The homogenates were centrifuged for 15 min at 13.000 rcf at 4°C in order to eliminate debris. Supernatants, containing extracted proteins, were precipitated using 13% TCA and 0.007% ß-mercaptoethanol in acetone, transferred to -20°C for 2 hrs and finally kept at 4°C for 2 hrs.

Samples were then centrifuged at 14000 rcf at 4°C for 15 min and the pellet was washed twice with cold acetone (100%), re-centrifuged at the same speed, mixed with 50-500 μL extraction buffer and centrifuged at 3000 rcf at 4°C for 25 min. Protein quantification was done by Bradford assay (BIO-RAD Hercules, CA) using Bovine serum albumin (BSA) as standard.

### Two-dimensional electrophoresis analysis

Two dimensional electrophoresis (2DE) analysis was performed combining IsoElectric Focusing (IEF) and Sodium Dodecyl Sulfate PolyAcrylamide Gel Electrophoresis (SDS–PAGE) as describe in [26,27]. Samples (1 mg) of protein were directly loaded by in-gel rehydration onto an IPG (Immobilized pH Gradient) gel strip for preparative analysis. IPG strips (18 cm, GE-Healthcare), with pH range 4-7, were rehydrated with 350 μL of IEF sample buffer (8 M urea, 2% w/v CHAPS, 40 mM DTT and 0.5% v/v IPG Buffer) containing the samples. Strips were covered with mineral oil and focusing was carried out in a IPGphor apparatus (GE-Healthcare) applying the following conditions: 12 h of rehydration at 30V, 1 h at 300 V (in gradient), 1 h at 300 V (step and hold), 3 h at 3500 V (in gradient), 3 h at 3500 V (step and hold), 3 h at 8000 V (in gradient) and a final step at 8000 V (step and hold until reached 50000 Vhs). After focusing, the strips were equilibrated, in two steps of 15 min (first step-equilibration buffer: 50 mM Tris–HCl, pH 8.8, 8 M urea, 30% v/v glycerol, 2% w/v SDS, 40 mM DTT; second step-equilibration buffer: in the same buffer in which DTT was replaced by 40 mM IAA). The second dimension, SDS-PAGE electrophoresis, was performed using BioRad Protean II XL (20×20 cm) vertical gel electrophoresis chambers. 12% polyacrylamide gels were run at 15ºC with a constant current of 40 mA per gel. Molecular weight standards in a range from 10 to 150 kDa were from BioRad. Proteins resolved by 2DE, were visualized by colloidal Coomassie brilliant-blue staining for both analytical and preparative analyses. For computer analysis, three gels per year were selected (total of six gels for two years) for each sample. The Brilliant Blue G-Colloidal Concentrate Coomassie (Sigma) staining for preparative analysis was performed according to manufacturer’s instructions.

### Image analysis and statistical analysis

High resolution (300 dpi) images were acquired using the ProXpress CCD camera system (Perkin Elmer). Computer-assisted 2D image analysis was done using Progenesis SameSpots vs 3.2.3 gel analysis software (NonLinear Dynamics) for three technical replicates for each biological condition (different years) from three independent experiments (see above). Protein apparent relative molecular mass (Mr) was estimated by comparison with molecular weight (MW) reference markers (Precision, Bio-Rad, Hercules, CA) and pI values assigned to detected spots by calibration as described in the GE-Healthcare guide lines. The amount of protein was expressed as spot volume, which was defined as the sum of optical density of all the pixels that make up the spot as detected by the software. Protein level increase/decrease was quantified comparing the spot volumes normalized as percentage of the total volume in all the spots present in the gel.

Spots were considered to represent differentially expressed proteins on the basis of their ANOVA values (q-value) and fold change as evaluated by the software. Image software automatically order spots on the base of these values. Post-test analysis (Tukey’s test) was performed on the basis of the ANOVA results, in order to identify specific correlations among the samples. The relevance of each spot in discriminating between samples from different places was evaluated by principal component analysis as software tool for different combinations of differentially expressed spots.

### Protein identification by MALDI-TOF and nLC-ESI-MS\MS

Protein spots of interest were excised from gels, reduced, alkylated, and digested overnight with bovine trypsin (Roche Diagnostics Corp.) as previously described by Shevchenko [28]. Aliquots of the supernatant (1 μL) were used for MS analysis. MS analysis was done using the dried-droplet technique, with α-cyano-4-hydroxycinnamic acid as a matrix. Mass spectra were obtained with a MALDI-TOF Voyager DE-STR from Applied Biosystems/MDS Sciex. Ions were generated by irradiation with a pulsed nitrogen laser (337 nm UV, pulse duration 3 ns, pulse rate 3 Hz), and positive ions were accelerated and detected in the reflector mode. Instrument settings were: accelerating 20˙000 V, grid 64%, guide wire 0%, delay time 200 ns, shots/spectrum 100, mass range 750-4000 Da and low mass gate 700 Da. Spectra were acquired via Voyager Control Panel 5.10 from Applied Biosystems. Once acquired, spectra were processed with Data Explorer 4.0 from Applied Biosystems and internally calibrated with trypsin autolysis products and matrix clusters. MALDI-TOF data led to extracted and manually curated peptide monoisotopic peak lists (deprived from trypsin and matrix clusters signals) that were searched, via in-house Mascot Server 2.2.07, against the target database as detailed below except for mass tolerance for monoisotopic data that was set to 50 ppm and significance threshold of p < 0.05 set for the probability based Mascot Mowse Score.

For ESI analysis, 5 μL of trypsin digested samples were injected in a capillary chromatographic system Agilent 1100 Series equipped with a Nano Pump, Iso Pump, Degaser and a 8 μL injection loop (Agilent). Peptide separations occurred on a 10-15 cm fused silica emitter (75 μm i.d., 360 μm o.d.; Proxeon Biosystems) used as analytical RP nano column. The emitter was packed in-house with a methanol slurry of reverse-phase, fully end-capped 3-μm ReproSil-Pur 120 C18-AQ resin (Dr. Maisch GmbH), using a pressurized “packing bomb” operated at 50-60 bars.

Mobile phases consisted of water with 2% acetonitrile, 0.1% formic acid (v/v; buffer A) and acetonitrile with 2% water, 0.1% formic acid (v/v; buffer B). A 55-min gradient from 8% to 80% buffer B at a constant flow rate of 200 nl/min was used for peptides separation.

Eluting peptides were ionized by a nanoelectrospray ion source (Proxeon Biosystems) and analyzed on an API QStar PULSAR (PE-Sciex) mass spectrometer. Analyses were performed in positive ion mode. The HV Potential was set up around 1.8-2.0 kV. Full scan mass spectra ranging from m/z 350 to 1600 Da were collected and for each MS spectrum the two most intense doubly and triply charged ions peaks were selected for fragmentation (MS/MS range from m/z 100 to 1600 Da).

MS/MS spectra data files from each chromatographic run were combined and converted to mgf files using Mascot.dll (version 1.6b27) through Analyst QS 1.1 (Applied Biosystems) and searched (via Mascot Daemon 2.2.2 and in-house Mascot Server 2.2.07), first against a custom contaminant database (trypsin and common keratins partly derived from the cRAP collection), unmatched signals were then searched against the UniProt_Complete Proteome_tuber 2012_07 (7679 sequences; 3339250 residues) database. Mass tolerance was set to 200 ppm and 0.3 Da for precursor and fragment ions respectively. Searches were performed with trypsin specificity, alkylation of cysteine by carbamidomethylation, and oxidation of methionine as fixed and variable modifications respectively; ion score cut-off set to 20; two missed cleavages were allowed for trypsin specificity; the quality of MS/MS identifications was manually checked. Proteins obtained without functional identification were then used for Protein Blast Analysis (UniprotKb blastp) performed with default settings.

### Total RNA extraction and real-time PCR analysis

Total RNA was extracted from pulverized samples as described [29]. This protocol was selected on the basis of its ability to remove contaminants from RNA extracted samples. Electrophoresis using 1% agarose gel was performed for all RNA samples to check for RNA integrity, followed by spectrophotometric quantification and quality control. RNA samples were then subjected to DNase treatment using a Turbo DNA-free kit (Ambion, USA) to remove possible DNA contamination. RNA was then reverse-trascribed using SuperScript® III Reverse Transcriptase kit (Life Technologies, UK) with random primers. Gene expression analysis was carried out using an ABI Prism 7300 sequence detection system (Applied Biosystems, USA) as described by [30]. Quantitative PCR was performed using 30 ng cDNA and iQ™ Sybr Green Supermix (BioRad laboratories), according to the manufacturer’s instructions. Two technical replicates were performed for each biological replicate (n=4).

Expression of *T. magnatum* (AF054901) 18S rRNA was used as a housekeeping gene. Relative expression levels were calculated using Genorm (*http://medgen.ugent.be/genorm/*). Primers were designed using Primer 3. The following primer list is reported with its specific *Tuber* gene identification code (in brakets Uniprot code of the relative protein):

GSTUM_00009270001 (D5GJY5), 5’-gcactggcaccactcctacc-3’(forward), 5’-gaagaaggtgcccccaaaac-3’ (reverse); GSTUM_00007439001 (D5GGN7), 5’-gaccaggaataccgcaccaa-3’ (forward), 5’-tcctcctcagccttgtgagc-3’ (reverse); GSTUM_00008874001 (D5GJ78), 5’-gcgccatcaaggatattgga-3’ (forward), 5’-aacaccagtggcgatgtcct-3’ (reverse); GSTUM_00003332001 (D5G9M7), 5’-tcctctcgctcgcctatgag-3’ (forward), 5’-aacttcgacgaggtccacca-3’ (reverse); GSTUM_00005271001 (D5GAF9), 5’-gttttgacacccgccgataa-3’ (forward), 5’-aaggttcctgcacccacaga-3’ (reverse); GSTUM_00000555001 (D5GC43), 5’-gttgaaaacgcacgcctctc-3’ (forward), 5’-gccctcatcctcgacaacac-3’ (reverse); GSTUM_00005237001 (D5GAC6), 5’-acctgtgcgattctggtgct-3’ (forward), 5’-atccgtaggctcgccaaaat-3’ (reverse); GSTUM_00006427001 (D5GE86), 5’-gaagccaatctcggaggtga-3’ (forward), 5’-aaaacggcttccggtgtctt-3’ (reverse); GSTUM_00001447001 (D5G5R4), 5’-gagctcctcggaaagcatca-3’ (forward), 5’-ccaggaagaggggtttgtcc-3’ (reverse); DQ223686.1 (Q1ACW3), 5’-gaaggctctccgctacgaca-3’ (forward), 5’-accgcaagccttgactttga-3’ (reverse); AF054901, 5’-actagggatcgggcgatgtt-3’ (forward), 5’-cagccttgcgaccatactcc-3’ (reverse).

## Abbreviations

2-DE: Two-dimensional gel electrophoresis; qPCR: Quantitative polymerase chain reaction; SSR: Simple sequence repeat; MS: Mass spectrometry; MALDI-TOF: Matrix-assisted laser desorption/ionization-Time-of-Flight; ESI: Electrospray ionization; NAD^+^ or NADP^+^: Nicotinamide adenine dinucleotide or nicotinamide adenine dinucleotide phosphate; TCA: Tricarboxylic acid; ROS: Reactive oxygen species; ER: Endoplasmic reticulum; GTP: Guanosine triphosphate; CFU: Colony forming unit; CHAPS: 3[(3-Cholamidopropyl)dimethylammonio]-propanesulfonic acid; DTT: Dithiothreitol; BSA: Bovine serum albumin; IEF: IsoElectric Focusing; SDS–PAGE: Sodium Dodecyl Sulfate PolyAcrylamide Gel Electrophoresis; IPG: Immobilized pH Gradient; IAA: Iodoacetamide; Mr: Relative molecular mass; MW: Molecular weight.

## Competing interests

The authors declare that they have no competing interests.

## Authors’ contributions

FV and VL carried out the proteins extraction and separation; EA performed MS analysis; AB designed MS experiments; MA designed image and statistical analysis and revised the manuscript; RB and AM revised the mycological aspects of the manuscript; AA drafted the manuscript. All authors read and approved the final manuscript.
